# Associations of vitamin D status, bone health and anthropometry, with gross motor development and performance of school-aged Indian children who were born at term with low birth weight

**DOI:** 10.1136/bmjopen-2015-009268

**Published:** 2016-01-08

**Authors:** Suzanne Filteau, Andrea M Rehman, Aisha Yousafzai, Reema Chugh, Manpreet Kaur, H P S Sachdev, Geeta Trilok-Kumar

**Affiliations:** 1 Faculty of Epidemiology and Population Health, London School of Hygiene & Tropical Medicine, London, UK; 2 Department of Paediatrics and Child Health, Aga Khan University, Karachi, Pakistan; 3 Institute of Home Economics, University of Delhi, Delhi, India; 4 Sitaram Bhartia Institute of Science and Research, Delhi, India

## Abstract

**Objectives:**

There is little information regarding motor development of children born at term with low birth weight (LBW), a group that constitutes a large proportion of children in South Asia. We used data from infancy and at school age from a LBW cohort to investigate children's motor performance using causal inference.

**Design:**

Cross-sectional follow-up study.

**Setting:**

Delhi, India.

**Participants:**

We recruited 912 children aged 5 years who had participated in a trial of vitamin D for term LBW infants in the first 6 months of life.

**Outcome measures:**

We focused on gross motor development, using the Ages and Stages Questionnaire (ASQ) gross motor scale and several measures of motor performance. We examined the effects on these of current anthropometry, vitamin D status and bone health, controlling for age, sex, season of interview, socioeconomic variables, early growth, recent morbidity, sun exposure and animal food intake.

**Results:**

In adjusted analyses, stunted children (height-for-age Z (HAZ) <−2) took longer to run 20 m (0.52 s, 95% CI 0.35 to 0.70; p<0.001) and had greater odds of a failing score on the ASQ (OR 3.00, 95% CI 1.41 to 6.38, p=0.004). Greater arm muscle area was associated with faster run time, and the ability to perform more stands and squats in 15 s. Poorer vitamin D status was associated with the ability to perform more stands and squats. Lower tibia ultrasound Z score was associated with greater hand grip strength. Early growth and current body mass index had no associations with motor outcomes.

**Conclusions:**

Current HAZ and arm muscle area showed the strongest associations with gross motor outcomes, likely due to a combination of simple physics and factors associated with stunting. The counterintuitive inverse associations of tibia health and vitamin D status with outcomes may require further research.

**Strengths and limitations of this study**

- The work included a large cohort of low birth weight term infants for whom we had detailed anthropometric and health data, both in infancy and when they were school aged.
- We used causal inference to estimate direct effects.
- We were limited in our choice of development outcomes feasible under the study conditions and investigated only gross motor outcomes.
- We did not have any controls born at normal birth weight for comparison.

## Introduction

Infancy and early childhood are key periods during which adverse environments can impair and appropriate interventions can improve not only child survival, health and growth, but also child development. Stressors or interventions in these early periods can have lifelong effects.^1^ This knowledge has led to the current focus on the first 1000 days from pregnancy to age 2 years as a sensitive period for interventions.^2^

India has a high burden of children aged under 5 years not meeting their developmental potential.^3^ Mitigation of risks for poor development, which include a high prevalence of nutrition-related risks, in the South Asian context, needs to be explored further. A recent systematic review showed that children with intrauterine growth retardation (IUGR), which is very common in South Asia,^4^ are at risk for developmental delays.^5^ Among the 11 studies that reported neurodevelopmental delay in the first 3 years of life, 10 found motor delay. Catch-up growth can mitigate the adverse effects of IUGR on intellectual and psychological performance,^6^ but there appears to be little information about catch-up growth and gross motor outcomes. Motor performance is of particular importance in societies where much employment relies on physical strength and performance.

Our group previously investigated, in the Delhi Infant Vitamin D Supplementation (DIVIDS) trial, whether vitamin D supplements given to low birth weight term infants from age 1 week to 6 months are a beneficial intervention within the first 1000 days; we showed that the supplements could improve child linear and weight growth but that they did not affect morbidity or cytokine production.^7^ ^8^ We recently followed up the children from the DIVIDS trial, when they were aged about 5 years, and found that small differences remained in the anthropometry between treatment groups but there were no differences in bone structure as measured by quantitative ultrasound (QUS) and no differences in several measures of gross motor development.^9^ Vitamin D status changes rapidly enough that supplements in infancy no longer affected vitamin D status of the 5-year-olds. However, early growth effects of the vitamin D supplements could potentially lead to benefits for motor development since there is evidence from a recent meta-analysis that linear growth of children before 2 years of age is associated with later cognitive and motor development.^10^ Additionally, current vitamin D status, through effects on bone^11^ and muscle,^12^ ^13^ and their interaction,^14^ could affect motor performance. Physical exercise can also increase bone density^15^ as well as improve motor performance. Sunlight exposure can be both a means of improving vitamin D status and, in some countries, a marker of outdoor play and physical activity in young children.^16^ We used the detailed original and follow-up DIVIDS databases to investigate further how vitamin D status, anthropometry, early growth, bone health, diet and sun exposure affect development. Since the DIVIDS study concerned vitamin D and the primary outcome of the follow-up study was linear growth, we focused on gross motor development and performance on the grounds that motor functions would likely be more closely related to anthropometry and bone health than would other domains of child development such as cognition, language or behaviour.

## Methods

### Participants

Parents of participants in the original DIVIDS trial (referred to as DIVIDS-1, registered at ClinicalTrials.gov NCT00415402) were contacted by phone or in person using contact details provided in DIVIDS-1. Of the original 2079 DIVIDS-1 children, 912 were followed up. The sample size of the follow-up study was adequate for the primary outcome of height-for-age Z (HAZ) score. Further details of design and power calculations have been published.^9^

### Procedures

Between November 2012 and January 2014, the parents and children were invited to the study office on specified days and were provided either transport or travel reimbursement. Study visits included questionnaires regarding sociodemographic factors, morbidity history, diet by dietary diversity questionnaire, sunlight exposure, detailed anthropometry, child motor development testing, QUS, body composition by deuterium dilution in a subset and examination by a doctor. Venous blood samples were collected in lithium heparin vacutainers (Becton Dickenson, India) for measurement of 25-hydroxyvitamin D (25OHD), processed within half an hour and stored at −80°C until batched analysis. Each visit took about half a day and several children were present on most days.

### Anthropometry and QUS

Detailed anthropometry was conducted for the study but the present analyses use only weight, height, mid-upper arm circumference (MUAC) and triceps skinfold thickness. All measurements were taken in triplicate, using standard methods,^17^ and medians were used in analyses. HAZ and body mass index-for-age (BMIZ) were calculated using the WHO standards.^18^ Mid-upper arm muscle area (AMA), calculated from MUAC and triceps skinfold using standard methods,^17^ was used as an indicator of lean mass since we had deuterium dilution measures on only a subset of children.^9^

Bone structure and strength were measurement by QUS (Sunlight Omnisense 7000 Bone Sonometer, Israel).^19^ Measurements were taken at the distal radius and mid-shaft tibia of the child's dominant hand side. We used the settings for Caucasian population for Z scores-for-age in analyses.

### General child health

Several child health indicators were assessed: whether the child had ever been in hospital, whether they had been to the doctor within the past month and whether they had experienced a range of symptoms within the past 3 days. These three measures were highly related so, in analyses, we chose to use recent morbidity, that is, within the past 3 days, since it was considered most likely to affect motor performance.

### Diet

The dietary diversity questionnaire was an adaptation of the Food and Agriculture Organization of the United Nations (FAO) questionnaire.^20^ We were interested in the habitual diet of individual children rather than of the whole population so, rather than use a 24 h recall, we used food frequency over the past month. In addition, we were interested in micronutrient-rich foods, so removed categories on starchy staples and sweets. This resulted in 10 food groups: legumes/nuts, dairy, eggs, meat/poultry, fish, antioxidant-rich fruits, other fruits, antioxidant-rich vegetables, other vegetables and fats/oils. For the present analysis we focused on animal food groups (dairy, eggs, meat/poultry, fish) because of their importance for development of young children.^21–23^ Each of these food groups was coded 0 if eaten fewer than three times/week and 1 if eaten at least three times/week. For analyses, the values were coded as 0, 1, or ≥2 of these food groups consumed at least three times/week.

### 25-hydroxyvitamin D

25OHD was measured in duplicate by radioimmunoassay using a kit from DiaSorin (Stillwater, Minnesota, USA). An external standard (Vitamin D External Quality Assessment Scheme, DEQAS) was included in each run and results for it were within acceptable limits. Inter-assay coefficient of variation for a pooled serum sample included in each run was 10%. 25OHD results were log-normally distributed so analyses used natural log-transformed data.

### Motor ability tests

Testing took place both within the clinic and in a grassy yard next to the clinic. For only those children aged under 5.5 years, the maximum for which the test is designed, the Ages and Stages Questionnaire, Second Edition (ASQ; http://agesandstages.com), was used to assess gross motor development. The ASQ has been previously used successfully for young Indian children.^24^ ^25^ Following a review and some initial testing, we found no changes to the test items were required. Postgraduate research fellows were trained by one of the investigators (AY). The trained assessors encouraged the children to perform each activity. A child was given a score of 10 if he or she was observed performing the activity, 5 if the mother reported the child sometimes performing the activity at home and 0 if the child was not yet able to perform the activity. For each age range, six activities (overlapping but not identical for different age groups) were assessed and a maximum score of 60 could be achieved. Total scores had very non-normal distributions, so we analysed only the proportion of children falling below the age-specific cut-offs as specified in the test instructions. We recognise the limitations of using cut-offs in populations for which they were not designed but were conducting only within-population comparisons.^24^

We used two additional simple motor performance tests: time to run a marked off distance of 20 m and the number of stands and squats the child could perform in 15 s. The child was given two tries and his or her best score was used. We measured grip strength for the child's dominant hand, using a custom-designed dynamometer; the best score of three tries was used.

### Statistical methods

Data were double entered into Access databases, cross-checked, cleaned and analysed using Stata V.13.1. There were considerable missing data for a variety of reasons: 327 ASQ results because only children under 5.5 years were tested, 90 grip strength results because the equipment arrived only after the first 90 children were tested, and 50 run times and 71 squats results because many of the youngest children were unable to perform these or refused to try, and it was often difficult to distinguish between these. Since age was the main factor associated with missing data and since age was already controlled for in analyses, we considered values both for children who were unable and those who refused to try the tests as missing. In addition, a few children were missing data for key covariates, so for each motor test, separately, we used results only from children with complete data.

**Figure 1:**
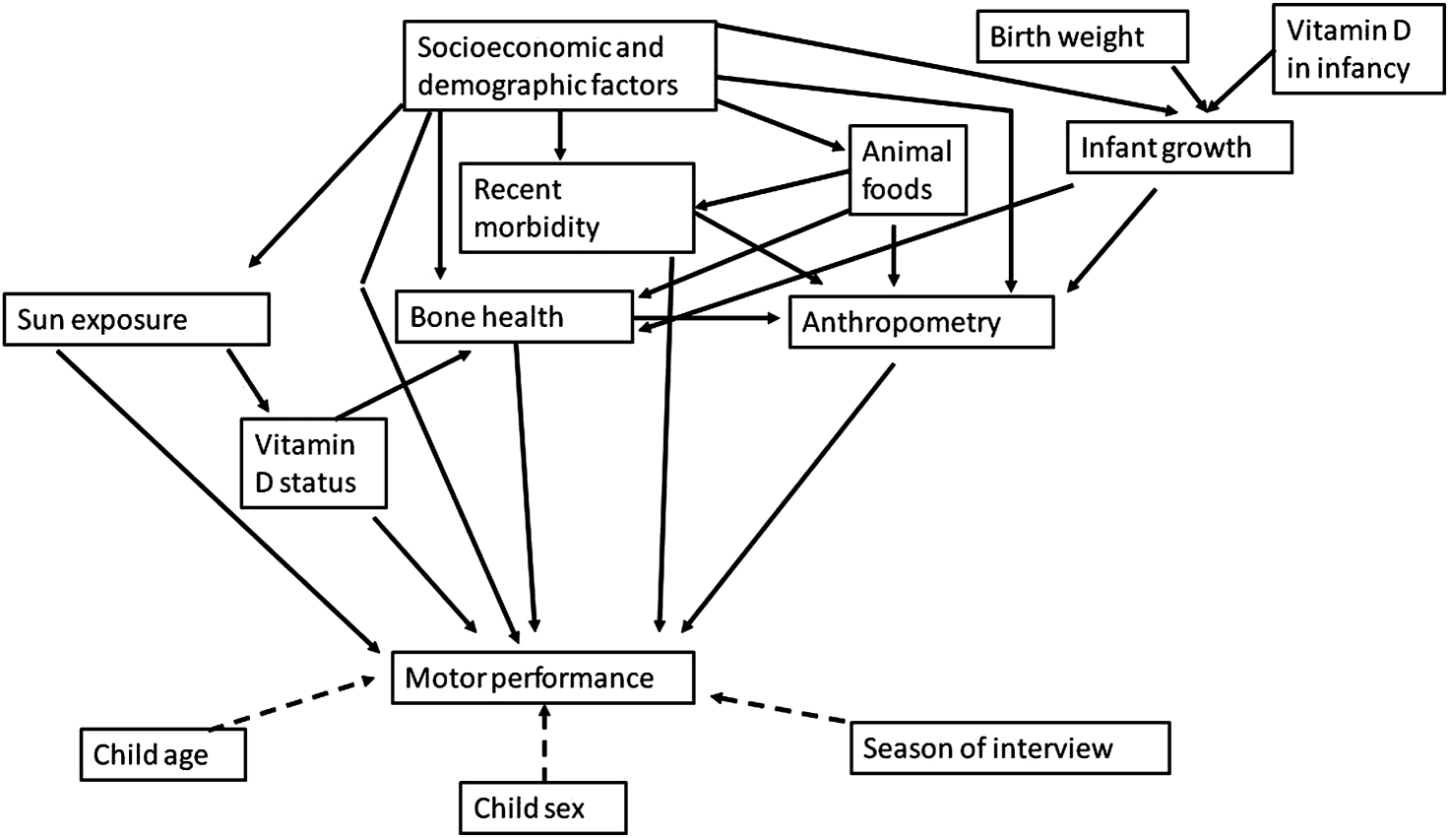
Conceptual framework for factors affecting motor development. Variables included under each of the group headings are: (1) motor performance: Ages and Stages Questionnaire pass/fail category, maximum grip strength, minimum run time, maximum number of squats in 15 s; (2) anthropometry: height and body mass index Z scores, arm muscle area; (3) vitamin D status: serum 25-hydroxyvitamin D; (4) bone health: radius and tibia quantitative ultrasound Z scores; (5) sun exposure: h/day; (6) diet: animal food groups; (7) infant growth: birth weight tertile, length-for-age Z score at 6 months, change in length-for-age Z score from birth to 6 months; (8) recent morbidity: reported symptoms in the past 3 days; (9) season of interview: 4-month divisions; (10) sociodemographic factors: quintiles from principle components analysis and (11) vitamin D in infancy: treatment group allocation in Delhi Infant Vitamin D Supplementation (DIVIDS)-1 trial.

Analyses were guided by the directed acyclic graph in figure 1, which focused on vitamin D status, anthropometry and bone health as well as probable contributors to these: socioeconomic status (SES) at follow-up, diet, recent morbidity, sun exposure and early growth. Analyses followed principles of causal inference^26^ and were aided by DAGitty, an online tool.^27^ SES was calculated using principal components analysis (PCA)^28^ and divided into quintiles; items offered into the PCA were: a list of assets, maternal and paternal education and occupation, and housing characteristics. Early growth was represented by three variables from the DIVIDS-1 database: birth weight divided into tertiles for the group followed up, tertiles of HAZ at age 6 months and tertiles of change in HAZ from birth to 6 months. Sun exposure was coded as never, <1 h/day, 1–2 h/day or >2 h/day. All analyses were controlled for child sex and age at follow-up. We also noticed an effect of season of testing on motor development outcomes. This was likely partly a true seasonal effect, for example, children being unable or unwilling to run fast outside in the very hot summer months in Delhi, and partly a function of the order we recalled children for testing since we first had sufficient funds to test only the youngest children but then received additional funding for testing more children. Therefore, we have controlled for season of testing, divided into three (March–June (summer), July–September (monsoon), October–February (winter)), for all analyses.

### Ethics

Parents provided written or thumbprint informed consent for their child's participation. Children were seen by a medical doctor and provided with treatments or referrals as required. Names and addresses were removed from data sets prior to analysis.

## Results

Table 1 describes sociodemographic characteristics, nutrition and health of the 912 children followed up. The average age was 5 years, and half the children were girls. Height, weight and BMI Z scores were all low, which reflects that the children were recruited as low birth weight term infants. Bone QUS scores were fairly normal and plasma 25OHD was low. Many children reported recent or previous illness.

**Table 1:** Characteristics of the 912 children followed up*

*Demographics*	
DIVIDS-1 treatment group, N (%) in the vitamin D arm	446 (49)
Age	
years (SD)	5.0 (1.0)
Sex N (%) female	478 (52)
Season of follow-up (N, %)	
March–June (Summer)	226 (25)
July–September (Monsoon)	215 (24)
October–February (Winter)	471 (52)
Family type (N, %)	
Nuclear	316 (35)
Joint	400 (44)
Extended	196 (21)
Mother's education (N, %)	
None	135 (15)
Primary	408 (45)
Secondary	288 (32)
College/university	81 (9)
Father's education (N, %)	
None	63 (7)
Primary	345 (38)
Secondary	400 (44)
College/university	104 (11)
Mother's occupation housewife (N, %)	893 (98)
Father's occupation (N, %)	
Unemployed/student	29 (3)
Unskilled	48 (5)
Semi-skilled	194 (21)
Skilled	512 (56)
Self-employed	115 (13)
Professional	14 (1.5%)
*Anthropometry and nutrition*	
Birth weight	
kg (SD)	2.22 (0.16)
Z score (SD)	−2.46 (0.45)
Birth length	
cm (SD)	45.7 (1.4)
Z score (SD)	−2.02 (0.77)
Length at 6 months	
cm (SD)	62.6 (2.2)
Z score (SD)	−1.87 (0.98)
Weight	
kg (SD)	14.3 (2.8)
Z score (SD)	−1.93 (0.95)
Height (n=911)	
cm (SD)	100.8 (8.2)
Z score (SD)	−1.82 (0.98)
Body mass index (n=911)	
kg/m^2^ (SD)	14.0 (1.2)
Z score (SD)	−1.10 (0.92)
Radius QUS (n=905)	
Z score (SD)	−0.64 (1.04)
Tibia QUS (n=906)	
Z score (SD)	−0.53 (1.01)
Plasma 25OHD (n=902)	
nmol/L (SD)	32.7 (23.0)
deficient (<25 nmol/L)	384 (43%)
borderline (25–50 nmol/L)	363 (40%)
sufficient (>50 nmol/L)	155 (17%)
Animal food groups at least 3 times/week†	
0 groups	153 (17%)
1 group	602 (66%)
≥2 groups	155 (17%)
*Health and sunlight exposure*	
Reported illness in the last 3 days‡ (N, %, n=906)	300 (33)
Visited the doctor in the last month (N, %, n=906)	286 (32)
Has ever stayed in hospital (N, %, n=906)	173 (19)
Sun exposure (N, %, n=911) (h/day)	
Never	62 (7)
<1	425 (47)
1–2	264 (29)
>2	160 (18)

*Where there was missing data, sample sizes are given in the left column.

†Animal food score is coded 0=none of dairy, eggs, meat, fish at least three times/week, 1=1 of these at least three times/week; 2=2 or more of these groups at least three times/week.

‡Diarrhoea, convulsions, cough, runny nose, fever, difficulty breathing, vomiting, eye problem, ear problem, skin rash, lethargy, other illness.

DIVIDS, Delhi Infant Vitamin D Supplementation; 25OHD, 25-hydroxyvitamin D; QUS, quantitative ultrasound.

Most children (93%) scored within the acceptable range for their age on the ASQ. The mean values for other outcomes were: maximum grip strength, 2.49 kg (n=830, SD 0.93), minimum time to run 20 m, 7.1 (n=861, SD 1.8) s and maximum number of squats within 15 s, 12 (n=840, SD 3).

Table 2 shows crude associations of key potential mediators—25OHD, anthropometry and bone health—and potential modifiers—anthropometry in infancy, sun exposure, animal food intake, SES and recent morbidity—with motor outcomes. Plasma 25OHD had no associations with the outcomes. Greater HAZ at follow-up, both as a continuous variable and comparing stunted with non-stunted children, was associated with faster run time and lower likelihood of failing on the ASQ. There was no association of current BMIZ with any of the outcomes. Greater AMA was associated with faster run time and more squats, and marginally associated with lower likelihood of failing on the ASQ. Regarding infant anthropometry and growth, birth weight showed no associations but both length at 6 months and length gain from birth to 6 months were associated with faster run speed; the highest length tertile at 6 months also had reduced likelihood of failing on the ASQ. Tibia QUS Z score was associated with faster running speed and lower grip strength; radius QUS Z score showed no associations. Neither sun exposure nor animal food intake was associated with any outcomes. SES quintiles showed non-linear associations with number of squats such that children in the second lowest quintile could perform the most. Recent morbidity was associated with increased run time.

**Table 2:** Crude associations of key variables with motor outcomes*

	Low motor ASQ score	Maximum grip strength (kg)	Minimum run time (s)	Maximum number of squats
N	560†	808	842	821
25-OH-vitamin D (log ng/mL)	0.80 (0.25 to 2.49) p=0.69	0.005 (−0.23 to 0.24) p=0.96	−0.27 (−0.59 to 0.05) p=0.10	−0.52 (−1.21 to 0.17) p=0.14
HAZ	**0.54 (0.38 to 0.78)** **p=0.001**	0.03 (−0.04 to 0.09) p=0.42	−**0.34 (−0.42 to −0.25)** **p<0.001**	−0.04 (−0.24 to 0.16) p=0.72
Height-for-age‡				
Z≥−2	**1**	0	**0**	0
Z<−2	**2.71 (1.31 to 5.61)** **p=0.007**	−0.06 (−0.19 to 0.07) p=0.37	**0.54 (0.37 to 0.72)** **p<0.001**	0.04 (−0.34 to 0.42) p=0.85
BMIZ	0.89 (0.58 to 1.37) p=0.60	0.04 (−0.03 to 0.11) p=0.22	−0.02 (−0.12 to 0.08) p=0.66	0.03 (−0.18 to 0.23) p=0.80
BMIZ‡				
Z≥−2	1	0	0	0
Z<−2	1.04 (0.34 to 3.18) p=0.95	−0.14 (−0.31 to 0.03) p=0.12	0.11 (−0.13 to −0.35) p=0.37	−0.01 (−0.53 to 0.50) p=0.96
Birth weight (tertile)				
Low <2.15 kg	1, p=0.16	0, p=0.17	0, p=0.17	0, p=0.91
Middle 2.15 to <2.31 kg	0.77 (0.36 to 1.66)	−0.07 (−0.22 to 0.09)	−0.16 (−0.31 to 0.06)	−0.10 (−0.57 to 0.37)
High ≥2.31 kg	0.42 (0.17 to 1.02)	0.08 (−0.08 to 0.23)	−0.20 (−0.41 to 0.02)	−0.06 (−0.52 to 0.40)
Length-for-age Z score at 6 months (tertile)§				
Low <−2.28	**1, p=0.01**	0, p=0.54	**0, p=0.005**	0, p=0.51
Middle −2.28 to −1.43	**1.74 (0.76 to 3.98)**	0.04 (−0.13 to 0.21)	**−0.14 (−0.36 to 0.09)**	−0.24 (−0.73 to 0.25)
High ≥−1.42	**0.27 (0.07 to 0.99)**	0.09 (−0.07 to 0.26)	**−0.37 (−0.59 to −0.14)**	0.006 (−0.48 to 0.49)
Length-for-age Z score gain 0–6 months (tertile)§				
Low <−0.17 gain	1, p=0.42	0, p=0.60	**0, p=0.03**	0, p=0.79
Middle −0.17 to 0.51 gain	2.06 (0.70 to 6.00)	−0.08 (−0.25 to 0.08)	**0.04 (−0.18 to 0.25)**	0.16 (−0.31 to 0.63)
High >0.51 gain	1.78 (0.60 to 5.28)	−0.04 (−0.20 to 0.13)	**−0.24 (−0.46 to −0.01)**	0.11 (−0.38 to 0.60)
Radius QUS Z	0.87 (0.63 to 1.19) p=0.37	−0.02 (−0.08 to 0.04) p=0.52	−0.07 (−0.16 to 0.02) p=0.11	0.12 (−0.06 to 0.30) p=0.20
Radius QUS Z‡				
Z≥0	1, p=0.18	0, p=0.67	0, p=0.07	0, p=0.25
0<Z≥−2	2.47 (0.84 to 7.24)	0.06 (−0.09 to 0.20)	0.23 (0.03 to 0.43)	−0.24 (−0.67 to 0.18)
Z<−2	1.30 (0.27 to 6.16)	0.09 (−0.16 to 0.35)	0.12 (−0.23 to 0.46)	−0.61 (−1.35 to 0.14)
Tibia QUS Z	1.20 (0.88 to 1.65) p=0.25	**−0.11 (−0.17 to −0.04)** **p=0.002**	**−0.09 (−0.18 to −0.003)** **p=0.04**	−0.05 (−0.24 to 0.15) p=0.62
Tibia QUS Z‡				
Z>=0	1, p=0.77	**0, p=0.004**	0, p=0.20	0, p=0.38
0<Z≥−2	0.79 (0.38 to 1. 64)	**0.19 (0.05 to 0.33)**	0.07 (−0.12 to 0.27)	0.17 (−0.25 to 0.59)
Z<−2	0.69 (0.18 to 2.61)	**0.42 (0.14 to 0.70)**	0.34 (−0.03 to 0.71)	−0.31 (−1.10 to 0.48)
Sun exposure (h/day)				
Never	1, p=0.06	0, p=0.52	0, p=0.14	0, p=0.22
<1	0.29 (0.11 to 0.79)	0.13 (−0.14 to 0.40)	−0.35 (−0.73 to 0.02)	0.48 (−0.33 to 1.29)
1–2	0.29 (0.10 to 0.81)	0.16 (−0.12 to 0.44)	−0.40 (−0.78 to −0.02)	0.41 (−0.42 to 1.24)
>2	0.36 (0.13 to 1.01)	0.22 (−0.08 to 0.51)	−0.47 (−0.87 to −0.07)	0.86 (−0.01 to 1.73)
Animal food intake score¶				
0	1, p=0.90	0, p=0.48	0, p=0.77	0, p=15
1	0.98 (0.38 to 2.54)	0.10 (−0.07 to 0.28)	−0.09 (−0.32 to 0.15)	−0.40 (−0.91 to 0.10)
≥2	0.80 (0.25 to 2.54)	0.05 (−0.17 to 0.27)	−0.06 (−0.36 to 0.23)	−0.63 (−1.27 to 0.02)
SES quintile				
Lowest	1, p=0.18	0, p=0.64	0, p=0.35	**0, p=0.008**
Low	0.38 (0.12 to 1.18)	−0.18 (−0.42 to 0.07)	−0.05 (−0.38 to 0.28)	**0.72 (0.01 to 1.43)**
Middle	0.30 (0.09 to 1.01)	−0.14 (−0.37 to 0.09)	−0.06 (−0.26 to 0.37)	**−0.32 (−0.99 to 0.30)**
High	0.36 (0.13 to 0.97)	−0.08 (−0.29 to 0.14)	−0.005 (−0.30 to 0.29)	**−0.27 (−0.91 to 0.37)**
Highest	0.57 (0.23 to 1.44)	−0.12 (−0.33 to 0.10)	−0.19 (−0.48 to 0.10)	**−0.27 (−0.90 to 0.37)**
Recent morbidity—yes	1.58 (0.80 to 3.10) p=0.19	−0.07 (−0.20 to 0.07) p=0.31	**0.30 (0.12 to 0.48)** **p=0.01**	−0.24 (−0.64 to 0.16) p=0.24

Bold typeface indicates significance at p<0.05.

*Values represent linear regression coefficients (grip strength, run time, squats) or ORs of scoring below the age-specific cut-off for ASQ, (95% CI), Wald p value, controlling for child sex, child age at follow-up testing in whole years and categorical season of follow-up testing.

†ASQ test performed only in children <5.5 years.

‡43% of children had HAZ<−2, 16% had BMIZ; for radius QUS 9% had Z<−2, 64% had −2<Z<0, 26% had Z>0; for tibias QUS 7% had Z<−2, 62% had −2<Z <0, 30% had Z>0.

§Sample sizes are reduced to n=411 for low motor ASQ score, n=696 for grip strength, n=727 for run time, n=709 for squats due to missing 6 month growth data.

¶Animal food score is coded 0=none of dairy, eggs, meat, fish at least three times/week, 1=1 of these at least three times/week; 2=2 or more of these groups at least three times/week.

ASQ, Ages and Stages Questionnaire; BMI, body mass index; BMIZ, BMI-for-age Z; HAZ, height-for-age Z; QUS, quantitative ultrasound; SES, socioeconomic status.

In view of these results, and because tibia and radius QUS Z scores were correlated (r=0.46, p<0.001), further analyses used HAZ to represent current anthropometry, AMA to represent lean body mass, length-for-age tertile at 6 months to represent early growth and tibia Z score to represent bone health.

We used the directed acyclic graph in figure 1 to investigate further the effects on motor function of the three proximal factors of key interest: current vitamin D status, anthropometry and bone health. The minimal set of potential confounders needed to determine the direct effect of vitamin D status included SES, sun exposure, HAZ, tibia Z score and recent morbidity. Control for these had little effect on associations between 25OHD and motor outcomes (comparing table 3 with table 2), except that, following adjustment, higher 25OHD was associated with fewer squats. The minimal set of potential confounders for HAZ and AMA included SES, tibia Z score, recent morbidity and 25OHD; and for tibia Z score, included SES, 25OHD, HAZ and recent morbidity; again adjustment in both cases had little influence on the results.

**Table 3:** Adjusted associations for estimating the direct effect of vitamin D status, height-for-age and bone health with motor outcomes*

	Low motor ASQ score	Maximum grip strength (kg)	Minimum run time (s)	Maximum number of squats
N	560	808	842	821
25-OHD (per unit increase in log ng/ml)†	0.86 (0.25 to 2.94) p=0.81	0.007 (−0.23 to 0.24) p=0.96	−0.20 (−0.52 to 0.11) p=0.20	**−0.75 (−1.45 to −0.05)** **p=0.04**
Height-for-age‡				
Z≥−2	**1**	0	**0**	0
Z<−2	**3.00 (1.41 to 6.38)** **p=0.004**	−0.07 (−0.20 to 0.06) p=0.30	**0.52 (0.35 to 0.70)** **p<0.001**	−0.02 (−0.40 to 0.37) p=0.94
Arm muscle area (per unit increase in cm^2^)‡	0.85 (0.70 to 1.02) p=0.08	0.02 (−0.006 to 0.05) p=0.12	**−0.08 (−0.11 to −0.04)** **p<0.001**	**0.10 (0.02 to 0.18)** **p=0.02**
Tibia QUS§				
Z≥0	1, p=0.77	**0, p=0.003**	0, p=0.30	0, p=0.39
0<QUS Z≥−2	0.83 (0.39 to 1.77)	**0.19 (0.05 to 0.33)**	0.09 (−0.10 to 0.27)	0.08 (−0.34 to 0.50)
QUS Z<−2	0.62 (0.16 to 2.43)	**0.42 (0.14 to 0.70)**	0.28 (−0.08 to 0.64)	−0.43 (−1.21 to 0.36)

Bold typeface indicates significance at p<0.05.

*Values represent linear regression coefficients (grip strength, run time, squats) or ORs of scoring below the age-specific cut-off (ASQ test done only for children <5.5 years), (95% CI), p value.

†Adjusted for age, sex, season of interview, sunlight exposure (coded 0, never; 1, <1 h/day; 2, 1–2 h/day; 3, >2 h/day), SES, recent morbidity, tibia Z score and HAZ score.

‡Adjusted for age, sex, season of interview, tibia Z score, SES, recent morbidity and 25OHD.

§Adjusted for age, sex, season of interview, 25OHD, SES, HAZ and recent morbidity.

ASQ, Ages and Stages Questionnaire; HAZ, height-for-age Z; 25OHD, 25-hydroxyvitamin D; QUS, quantitative ultrasound; SES, socioeconomic status.

## Discussion

HAZ and AMA showed the strongest associations with motor outcomes. Interestingly, BMI was not associated with motor performance among this population of children with rather low BMI. This may reflect the fact that BMI includes both lean and fat mass and it is the lean component that primarily affects motor performance. In general, current vitamin D status was not associated with better motor performance. In adjusted analyses only, 25OHD was inversely associated with the number of squats children could perform; we believe this may be a chance finding resulting, in part, from statistical control of the complex interactions among vitamin D status, season of interview, sun exposure, SES and physical activity. Lower SES was associated with ability to perform more squats; we did not investigate mechanisms for this but it is possible that poorer children are more used to squatting, for example, because of their living conditions. Season of year was highly associated with vitamin D status, as expected, and, due to financial constraints, children were sampled in a way that season of year was also highly associated with age at follow-up. These complex interactions would have made estimating direct causal effects challenging. Bone health, as measured by QUS, was also associated with motor performance: higher tibia Z score was associated with faster run time which is plausible. However, the association of higher tibia z score with lower grip strength is counter-intuitive both because of the inverse association and because grip strength is a function of arm, not leg, muscle and bone. We are unable to explain the tibia bone and grip strength relationship so, in spite of the low p value, it is possible that the association was due to chance.

The association of greater height and AMA with faster running speed could be due primarily to simple physics, that is, a larger child may have longer or more muscular limbs. However, height was also associated with the ASQ, which does not simply measure current performance alone, but development of skills as well, so other mechanisms are likely involved. Poor linear growth is a marker for many types of stresses in early life—nutritional, environmental and social—which could affect development. Other studies have found associations between low HAZ and cognitive as well as motor outcomes.^10^ ^29^ ^30^ Other potential mechanisms for associations between child growth, and both motor and cognitive development, include a smaller, less healthy child being less active and exploring their environment less, or appearing younger and thus being treated as such by adults.^31^

Vitamin D, through its well-established role in bone growth and health, or through direct effects on muscle physiology,^13^ could potentially have influenced motor development, although we found limited associations. Interactions between vitamin D and motor development are likely complex, as suggested by a recent trial of vitamin D supplements in infants, which found better gross motor achievement in infants given a requirement level, compared to higher levels, of vitamin D.^32^ Others have shown an interaction between vitamin D status, anthropometry, bone mineral content and physical activity, on physical performance of adolescents.^33^ In our study, we were unable to measure physical activity but did measure sun exposure, which, in some populations, correlates with physical activity in children^16^; however, neither sun exposure nor vitamin D status was associated with motor outcomes.

Previous studies of associations of height with child cognitive development have investigated whether it was growth during specific periods or attained height that was key. The multicentre Young Lives study found that low HAZ at 8 years of age was associated with poorer cognitive function and school performance at that age, indicating the importance of attained height, but also that catch-up in HAZ between 1 and 8 years of age was associated with better performance.^29^ There were also benefits in HAZ from performing catch-up between ages 8 and 15 years.^34^ A multicentre study of five birth cohorts found that conditional weight gain, a measure of catch-up gain or loss over time, was associated with schooling outcomes, especially among children born in the lowest birth weight tertile.^35^ In our study, early growth had limited association with motor outcomes. Together, these studies suggest that it is attained height, irrespective of when the growth occurred, that is the most important factor for motor outcomes. This result supports continued promotion of a good diet and other health interventions even past the first 1000 days, for example, during adolescence, which is a sensitive time in the life course for bone development.^33^

There have been few studies on the development of children born with IUGR—most of these are in high-income countries, and none in South Asia, where low birth weight is common.^5^ ^24^ ^31^ In Iranian 5-year-olds, mean scores in all ASQ domains were significantly lower in low birth weight children when compared to their normal birth weight controls.^36^ The DIVIDS cohort included no normal birth weight children so we are unable to compare the children's performance directly against such controls; nevertheless, only 7% failures on the ASQ suggest serious impairments were rare. A previous study using the ASQ for younger Indian children, aged 4–24 months, and of whom 80/200 were considered at high risk of poor development, found 30% failed the gross motor item on the ASQ.^25^ Among Indian children, aged 6–30 months, from a similar community to the DIVIDS cohort, the mean score on the ASQ was 46.2 (SD 14.1); we found a similar mean of 53.9 (SD 9.0), although we did not use the continuous results in analyses because of the non-normal distribution. The similarity in performance of the two cohorts may in part be because the gross motor subscale seemed insensitive, with all participants being able to perform many items.^24^

Strengths of our study include its fairly large sample size and its rich database, including early growth data, which permitted investigation of many interacting factors that could affect motor performance of young children born at term with low birth weight. Limitations include the motor development tests, which were feasible under the study conditions. We focused on gross motor development because the main planned study outcome was growth, so we lack information about other domains of child development. However, there is little theoretical support for effects of vitamin D supplements on cognitive, language or social-emotional development in the absence of effects on motor development. The ASQ is designed to identify children developing abnormally^37^ and may not be sensitive enough to detect small effects of the factors of interest here. However, available tests that are more sensitive to nutritional differences, for example, the Bayley Scales of Infant Development,^38^ are more appropriate for younger children than those in our cohort. Running speed appeared the most sensitive motor performance measure, but measures only one component of motor function. Grip strength is increasingly used as an indicator of health or frailty in populations at risk for poor health,^39–41^ and has been recently shown to be associated with morbidity and mortality in a multinational study of adults.^42^ However, it is unclear exactly what grip strength measures since it is not specifically muscle mass or illness.^43^ We were unable to measure children's regular physical activity, which is an important contributor to gross motor performance.

In summary, height and AMA were the factors most strongly associated with motor performance of children born at term with low birth weight and aged about 5 years at testing. This was independent of factors that contribute to attained height and AMA such as early growth, animal food intake and SES, suggesting the main mechanism is through simple physics. Our results provide no evidence to support associations of vitamin D on motor development or performance that are independent of height. The association of current height with performance, previous studies showing the benefit for cognitive development of catch-up height growth at any time,^29^ ^34^ ^35^ and interactive benefits of vitamin D status and physical activity on motor performance of adolescents,^33^ support ongoing efforts to improve child nutrition and activity even after the first 1000 days.
